# Changes in predicted protein disorder tendency may contribute to disease risk

**DOI:** 10.1186/1471-2164-12-S5-S2

**Published:** 2011-12-23

**Authors:** Yang Hu, Yunlong Liu, Jeesun Jung, A Keith Dunker, Yadong Wang

**Affiliations:** 1 Center for Computational Biology and Bioinformatics, Indiana University School of Medicine, Indianapolis, IN, USA; 2 School of Computer Science and Technology, Harbin Institute of Technology, Harbin, China; 3 Department of Medical and Molecular Genetics, Indiana University School of Medicine, Indianapolis, IN, USA; 4 Center for Medical Genomics, Indiana University School of Medicine, Indianapolis, IN, USA; 5 Department of Biochemistry and Molecular Biology, Indiana University School of Medicine, Indianapolis, IN, USA

## Abstract

**Background:**

Recent studies suggest that many proteins or regions of proteins lack 3D structure. Defined as intrinsically disordered proteins, these proteins/peptides are functionally important. Recent advances in next generation sequencing technologies enable genome-wide identification of novel nucleotide variations in a specific population or cohort.

**Results:**

Using the exonic single nucleotide variations (SNVs) identified in the 1,000 Genomes Project and distributed by the Genetic Analysis Workshop 17, we systematically analysed the genetic and predicted disorder potential features of the non-synonymous variations. The result of experiments suggests that a significant change in the tendency of a protein region to be structured or disordered caused by SNVs may lead to malfunction of such a protein and contribute to disease risk.

**Conclusions:**

After validation with functional SNVs on the traits distributed by GAW17, we conclude that it is valuable to consider structure/disorder tendencies while prioritizing and predicting mechanistic effects arising from novel genetic variations.

## Background

"Sequence → Structure → Function" is the traditional view that amino acid sequences determine the structure of a protein molecule and that a definite protein structure is a prerequisite to biological function. This view has been amended by the finding that more and more proteins possess no definite ordered three-dimensional structure but are still involved in key biological processes, including cell cycle and gene regulation, molecular recognition, assembly of complexes, and signalling in general [1,2]. Indeed, over 33% of eukaryotic proteins contain structure-lacking regions. This kind of protein is often named "intrinsically disordered proteins" (IDPs). Several studies have shown a strong correlation between disease-associated proteins and proteins containing significant amounts of intrinsic disorder [3], leading to the D2 concept of "disorder in disease" [4]. Complex diseases such as cancer, neurodegenerative diseases, cardiovascular diseases, and diabetes are often associated with IDPs [3], likely because errors in signalling and regulation arising from IDPs are important for these disease associations. It was found that mutations that cause disorder tendencies to flip to structure tendencies are the most likely mutations in disordered regions to be disease-causing [5].

Recent advances in genetic studies enabled the discovery of many genetic regions linked or associated to complex diseases, using array-based genotyping technology, or more recently, next generation sequencing technology. Many known or novel single nucleotide variations have been identified, and their potential roles on disease pathogenesis are unknown. Many bioinformatics tools including FastSNP [6], Panther [7], PolyPhen2 [8], SIFT [9], SNPs3D [10] and SPOT [11], have been developed; many of them prioritize the SNV functions based on their roles in affecting protein structures.

Of particular interest here is that one study reported that 114 out of 122 (93%) single amino acid polymorphisms (SAPs) located in disordered regions are associated with disease. Thus SAPs occurring in disordered regions are highly likely to affect the functions of the proteins and be associated with disease [12].

In the present study, we systematically evaluate the potential disease risk on the SNVs whose resultant amino acid changes, SAPs, can change their structure/disorder tendencies, based on the single nucleotide variants derived from the 1,000 Genome Project and distributed by the Genetic Analysis Workshop (GAW17).

## Methods

**Figure 1:**
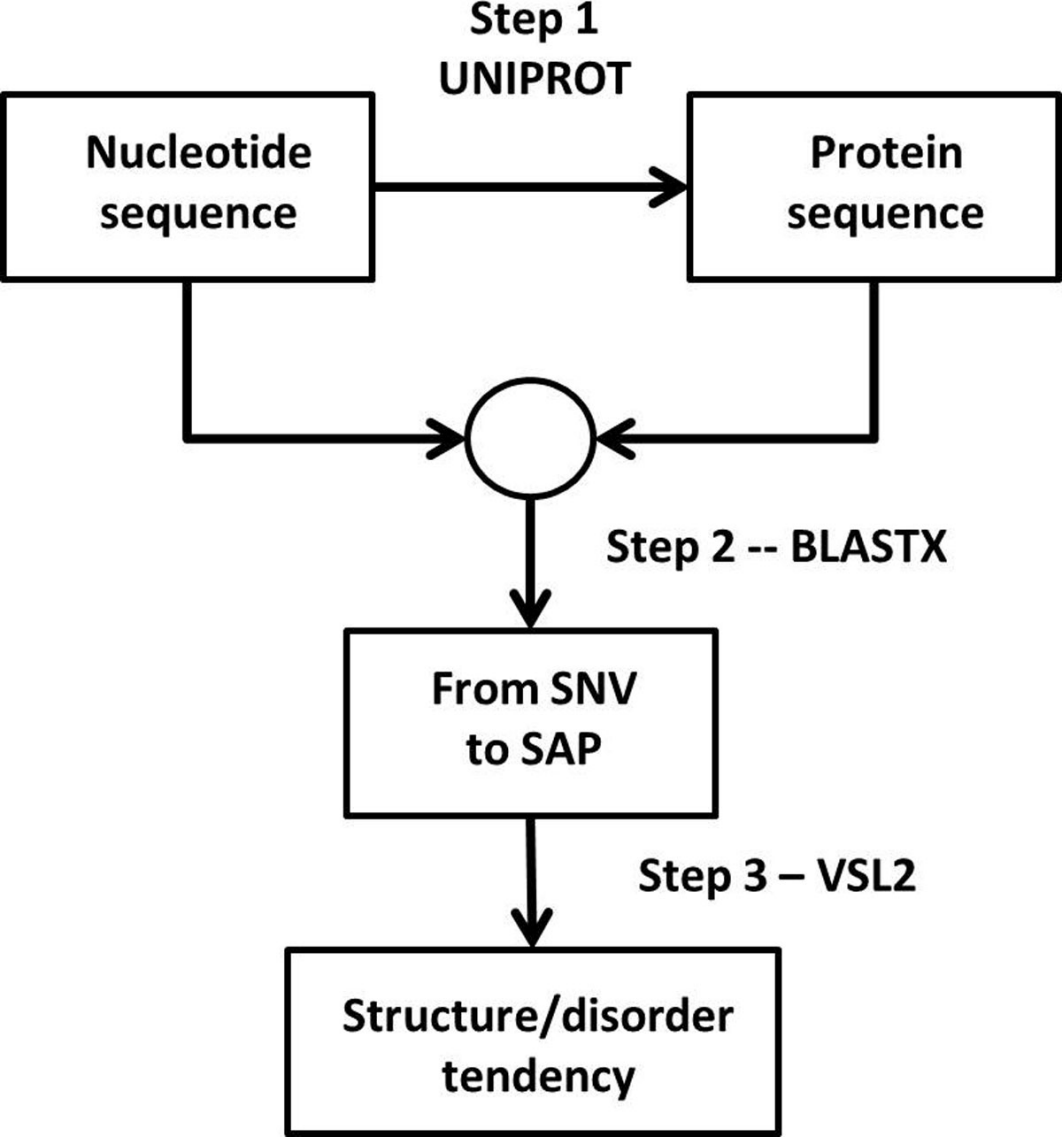
**Schematics of the workflow**.

The overall analysis workflow includes three major steps (Figure 1): 1. retrieving protein sequences for the genes of interest; 2. mapping genetic variations in nucleotide level into proteomic variations in amino acid level; and 3. assessing the capability of a genetic variation to change the disorder/structured tendencies. This workflow integrates different levels of bioinformatics approaches, and allows understanding how single nucleotide variations can change the predicted structure/disorder estimate of the stability of the protein structures, and how these changes affect disease risk.

### Dataset

SNV genotypes for 697 individuals were obtained from the sequence alignment files identified in the 1,000 Genomes Project and distributed by the Genetic Analysis Workshop (GAW17). A total of 24,487 exonic SNVs within 3205 autosomal genes were included in the data, regardless whether they are synonymous or non-synonymous. In this study, we focus our analysis on the missense non-synonymous variations that can cause single amino acid polymorphisms.

Three quantitative phenotypes (i.e. Q1, Q2, Q4) are generated for each of the unrelated individuals, and a total of 200 simulations are available to us. The disease model of Q1 includes 39 SNPs in 9 genes from VEGF pathway; Q2 is influenced by 72 SNPs in 13 genes related to cardiovascular risk and inflammation; Q4 is not affected by any of the available SNPs. Liability to disease is defined as latent liability+Q1+Q2-Q4, where latent liability is determined by 51 SNPs in 15 genes involved in the VEGF pathway.

### Retrieving protein sequences

Since UniProtKB/Swiss-Prot is a high quality manually annotated and non-redundant protein sequence database [13], we choose UniProtKB/Swiss-Prot to retrieve protein sequences. Furthermore, for every gene in the list, we retrieved both the canonical sequence and isoform data, downloaded from UniProtKB/Swiss-Prot database. We used the gene symbol in the GAW17 data set as the query to search for the amino acid sequences. Among the 3,205 genes provided in the data set, amino acid sequences of 2,893 genes were available in the UniProtKB/Swiss-Prot database, and retrieved for further analysis. Among the 24,487 SNVs provided in the GAW 17 data, 18,075 SNVs were mapped in the UniProtKB/Swiss-Prot database [13]. We exclude synonymous and nonsense non-synonymous mutations from further analysis, since the former causes no change in disorder score and the latter leads to truncation of the protein.

### Converting genetic variation to proteomic variation

In order to map the single nucleotide variations into the appropriate amino acid sequences, we used BLASTX algorithm [14] downloaded from the National Center for Biotechnology Information. BLASTX program translates the query nucleotide sequences in all six possible reading frames and provides combined significance statistics for hits to different frames. The nucleotide sequences from human genome build 36 (hg18) were used to retrieve the nucleotide query for each gene, and the reference sequences were the amino acid sequences from UniProtKB/Swiss-Prot. For ~10% of the genes, one segment of amino acid sequence may be mapped to different parts of nucleotide coding regions. To ensure the accuracy of the mapping, we further confirmed that paired segments in the query nucleotide sequence and the resultant amino acid sequences are in the same sequential order. We found this dramatically increase the mapping accuracy.

### Assessing the capability of a genetic variation to change disorder/structured status

To evaluate the potential of an SNV to alter the disorder or structure tendency, we first predict the disorder score for a SNV in the specific sequence. In this study, the per-residue disorder predictor PONDR-VSL2 [15] was used. VSL2 is composed of a set of support vector machines trained on datasets containing structured and disordered regions of various lengths. VSL2 provides one disorder prediction score between 0 and 1 for each residual. A score above or below 0.5 indicates that the target amino acid is located in a predicted region of disorder or structure, respectively. Overall, VSL2 achieves accuracy close to 80% correct and is one of the more accurate disorder predictors currently available. Although PONDR-VSL2 used in our study is one of the most accurate protein disorder predictors. There still exist two sources of uncertainty in disorder prediction, one is model uncertainty, and the other one is data uncertainty. Such work has been discussed in recent study [16].

We then use the following strategy to calculate the ability of one SNV to change the tendency of a local sequence region to form disorder or structure:

(1) $$\Delta DS=DS_{min}-DS_{maj}$$

where *DS*_*min *_and *DS*_*maj *_represent the SNV's disorder scores for minor allele and major allele, respectively. A positive or negative Δ*DS *value indicates that the minor allele will be associated with an increased or decreased disorder potential, respectively.

## Results and discussion

### Disorder- and Structure-potential in SNVs

The hypothesis that we want to test is that SNVs that cause changes in the structure/disorder tendency are deleterious. Destabilization of a structured protein domain by a mutation is often harmful [17]. A positive Δ*DS *in a structured protein corresponds to this case. As indicated above 114/122 mutations in disordered regions were associated with disease [12], so we also need to consider the possibility that SNVs causing negative Δ*DS *might also be deleterious. Of course for a mutation, it would likely be the change in the structure ←→ disorder tendency,, Δ*DS *that would be important, not necessarily the absolute value.

### Context-dependence of Δ*DS *values

Using the VSL2 software [15], we evaluated how a given amino acid change alters the potential for protein disorder by calculating the Δ*DS *as described above. The output of the VSL2 software depends not only on the amino acid at a given position but also on the amino acids surrounding that position. Thus, a particular Δ*DS *value will depend both on the amino acid change and also on the sequence context of the given amino acid change. Here we will compare two scenarios: when the same amino acid substitution occurs in two isoforms of related proteins and when the same amino acid substitution occurs in unrelated proteins.

To determine context-dependence for different isoforms, we identified SNVs in 1,170 genes that have isoform records in UniProtKB/Swiss-Prot database. In total 1,082 SNVs were found to potentially exist in 3,229 different isoforms. Pair wise comparison of all of the related isoforms yields a distribution showing how much each Δ*DS *value changes as the sequence-context changes in a different isoform. This distribution shows a very strong peak very close to a shift of 0.0003 in the Δ*DS *value followed by an extended tail, giving a average of ~ 0.01 for the data. Such a small context-dependent change for most of the sequences likely results from the very similar amino acid sequences of the different isoforms. Given such a small context-dependent change, it is not surprizing that only 0.2% of these amino acid substitutions lead to a change in the sign of the Δ*DS *value.

**Figure 2:**
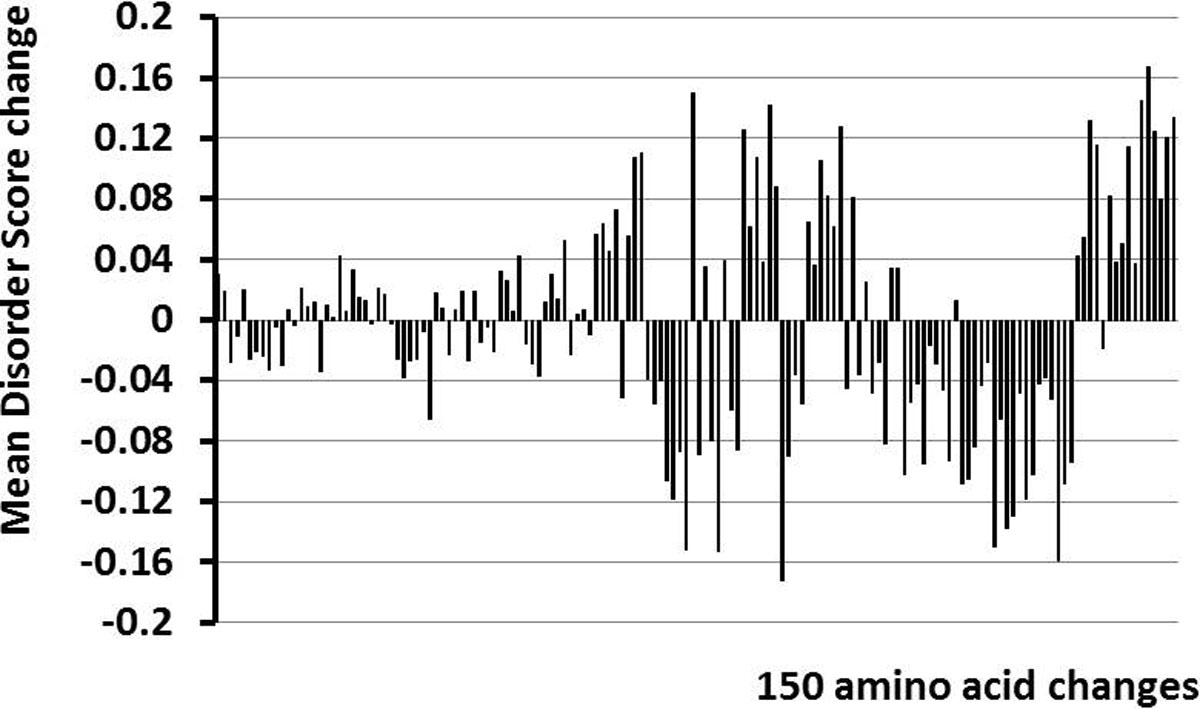
**Comparisons of average Δ*DS *scores changes**. The Δ*DS *values the same amino acid change in the different proteins were collected and averaged. Shown are the results for all 150 different amino acid changes.

Determining the context dependence for the same amino acid change in different sequences is more involved than the comparison of isoforms. Overall, there are 380 possible non-synonymous amino acid changes. However, if only one base change is allowed per codon, then there are only150 possible changes. These 150 possible changes were all observed in the dataset. The average Δ*DS *values, determined from the multiple instances of the same amino acid change, range from close to zero to slightly more than 0.16 (Figure 2).

The amino changes giving the 10 largest values for + or - average Δ*DS *are given in Tables 1, 2, while the codon changes are given in Tables 3, 4. An amino change might have one or more than one codon changes. The changes with large + average Δ*DS *correspond to mutating a large, hydrophobic residue (such as W, F, I, Y) into a hydrophilic one (S, N) or even into one with an electrical charge (R, D, K). This would certainly cause a shift towards a greater tendency to be disordered. On the other hand, the changes with large - Δ*DS *correspond to just the reverse, with a charged (K, E, R, D) or hydrophilic (S, G) residue mutating into a structure-promoting one (I, W, V, F, Y or C). This sort of change identifies a shift towards a greater structure tendency. The rather large standard deviations indicate significant contributions from the surrounding sequences.

**Table 1:** Rank of top 10 amino acid (AA) changes with + mean Δ*DS*

AA changes	Mean Δ*DS*	STD
W → S	0.17	± 0.08
F→ S	0.15	± 0.07
W→ R	0.14	± 0.07
I→ S	0.14	± 0.08
Y→ S	0.13	± 0.07
V → D	0.13	± 0.02
L→ S	0.13	± 0.06
I→ K	0.13	± 0.07
Y→ D	0.12	± 0.03
Y→ N	0.12	± 0.03

**Table 2:** Rank of top 10 amino acid (AA) changes with **- **mean

AA changes	Mean Δ*DS*	STD
K→I	-0.17	± 0.03
S→W	-0.16	± 0.08
G→W	-0.15	± 0.07
E→V	-0.15	± 0.05
R→W	-0.15	± 0.07
S→C	-0.14	± 0.06
S→F	-0.13	± 0.07
D→Y	-0.12	± 0.06
S→I	-0.12	± 0.07
S→Y	-0.11	± 0.07

**Table 3:** Rank of top 10 codon changes with + mean Δ*DS*

Codon changes	Mean Δ*DS*	STD
TGG→ AGG	0.23	± 0.002
ATT →AGT	0.18	± 0.038
TAC→ AAC	0.17	± 0.073
TGG→TCG	0.17	± 0.079
TTT→ TCT	0.16	± 0.086
TTA→ TCA	0.15	± 0.059
GTA→ GAA	0.15	± 0.080
TGC→ AGC	0.14	± 0.168
TAT→ TCT	0.14	± 0.065
TTC→ TCC	0.14	± 0.060

**Table 4:** Rank of top 10 codon changes with - mean Δ*DS*

Codon changes	Mean	STD
AGT→TGT	-0.20	± 0.040
AAA→ATA	-0.17	± 0.034
GAA→GTA	-0.17	± 0.034
TCG→TGG	-0.16	± 0.081
TCT→TAT	-0.16	± 0.044
AGG→TGG	-0.16	± 0.070
AGT →ATT	-0.16	± 0.064
GGG→TGG	-0.15	± 0.070
CGG→TGG	-0.15	± 0.073
GAG→GTG	-0.14	± 0.055

As expected from the large standard deviations in Tables 1, 2, pairwise comparisons of the same amino acid changes in different sequences give a very broad distribution. This distribution has a very weak peak near 0.006 and mean value of ~ 0.036. Large sequence differences for unrelated proteins accounts for these much more significant context-dependent changes here for the Δ*DS *values for a particular type of amino acid change as compared to the much smaller changes observed for the protein isoform data as discussed above.

### Minor allele frequencies and Δ*DS*

To illustrate the disorder/structured potential of SNVs, we focused our analysis on 10,254 missense non-synonymous SNVs, with4,345 and 5,909 showing positive and negative, Δ*DS *respectively.

Small values of Δ*DS *are expected to be less important, so partitioning the data into subsets having larger and smaller Δ*DS *values should be helpful. Examination of Figure 2 suggests that a threshold of |Δ*DS*| ≥ 0.04 would eliminate most of the small peaks in the figure. This value is also slightly larger than the mean value of ~ 0.036 for the context-dependent shifts of the Δ*DS *distribution as mentioned above. So, for both of these reasons, 0.04 was chosen as a threshold for our initial studies.

The positive and negative Δ*DS *sets were divided into subsets according to the magnitude of the Δ*DS *values (Table 5). As indicated, there are 1,572 SNVs with Δ*DS *≥ +0.04 and 2,629 SNVs with Δ*DS *≤ -0.04 (Table 5). The subsets with }Δ*DS*} ≥ 0.04 contain [(1,572 + 2,629)/10,254] →40% the data.

**Table 5:** Number of SNVs in synonymous/non-synonymous region

		Missense Non-synonymous		
			
	Synonymous	|Δ*DS*| > 0.04	|Δ*DS*| > 0	Nonsense Non-synonymous
+Δ*DS* Δ*DS*	--	1,572	4,345	--
	--	2,629	5,909	--

Total	7,511	--	10,254	310

**Figure 3:**
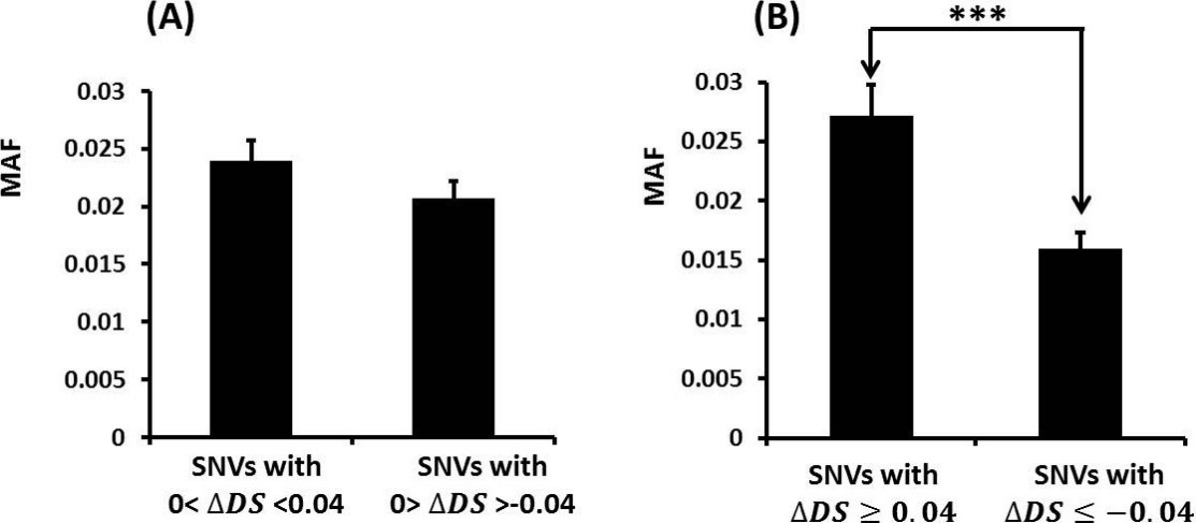
**Average MAFs for SNVs with different Δ*DS *values**. (A) Comparison of average MAFs for SNVs with |Δ*DS*| smaller than 0.04. (B) Comparison of average MAFs for SNVs with |Δ*DS*| greater than 0.04. *** stands for p-value<0.001

Next, the differences of the minor allele frequency (MAF) were compared for various subsets partitioned by their Δ*DS *scores. The MAF is the frequency of the SNV's less frequent allele in a given population. When Δ*DS *values are positive or negative but close to zero (Figure 3A), the two populations show similar MAF values as expected. The two populations are distinguishable, but just barely (with a p-value of only 0.163). On the other hand, as the Δ*DS *values become larger (Figure 3B), the MAFs of the two populations become more clearly distinct (p-value 1.58 × 10^-4^). To observe this significant change in the two populations for such a small change in Δ*DS *threshold value is remarkable and suggests that a change in the structure/disorder potential is indeed an important variable with regard to evaluating single amino acid changes in proteins.

### The effect of disorder/structured potential SNVs to protein and disease

Three traits namely Q1, Q2 and disease liability distributed by GAW17 are simulated to be affected by a particular set of non-synonymous SNVs within 9, 13 and 15 genes [18]. We further checked whether these genes also have SNVs that give significant changes (e.g. with |Δ*DS*| ≥ 0.04). Two, seven and six SNVs are found to have significant changes in the structure/disorder potential in trait Q1, Q2 and disease liability, respectively(Table 6).

**Table 6:** Summary of results on GAW17 simulated data

Trait	# of genes	# of SNVs with |Δ*DS*| > 0.04	# of SNVs with |Δ*DS*| > 0.04 in the answer sheet	# of SNVs in the answer sheet
Q1	9	8	2	39
Q2	13	12	7	72
disease liability	15	10	6	51

The details for these 2 + 7 + 6 = 15 examples are given in Table 7. In addition to the Δ*DS *values, also included are the reported amino change and the prediction score for the major allele.

**Table 7:** SNVs with |ΔDS |>0.04 in Q1, Q2 and disease liability

trait	SNV name	Gene name	AA changes	Δ*DS*	Major Allele Score
Q1	C19S4815	HIF3A	R→C	-0.10	0.81
Q1	C4S1879	KDR	V →M	0.04	0.11
Q2	C9S377	VLDLR	W→ C	0.18	0.51
Q2	C9S444	VLDLR	D→ Y	-0.16	0.36
Q2	C10S3050	SIRT1	P → L	-0.16	0.33
Q2	C8S476	LPL	I→S	0.15	0.17
Q2	C8S530	LPL	V→ G	0.13	0.32
Q2	C6S5446	VNN3	V→ I	-0.05	0.44
Q2	C8S442	LPL	D→N	-0.04	0.29
disease Liability	C17S4581	PRKCA	V → E	0.19	0.37
disease Liability	C18S2492	PIK3C3	V→ G	0.13	0.32
disease Liability	C1S9266	PIK3C2B	S → F	-0.13	0.77
disease Liability	C8S900	PTK2B	S → L	-0.13	0.38
disease Liability	C2S2307	BCL2L11	M → R	0.07	0.44
disease Liability	C1S9267	PIK3C2B	P → L	-0.06	0.83

In a previous study of deleterious mutations in structured proteins, energy calculations on 3-D structures showed that the deleterious mutations were those that destabilized the structure [17]. A + Δ*DS *for a region of protein predicted to be structured (e.g. with a prediction score < 0.5) would correspond to the potential destabilization of a region 3-D structure. Interestingly, 6/7 of the examples with + Δ*DS *values have scores < 0.5, with the remaining example having a score = 0.51. It would be interesting to try this approach on real data to determine if this approach could substitute for that described previously [17] for the identification of deleterious mutations in structured proteins. The advantage here is that the 3-D structures would not be needed.

Of the 15 examples, 8 involved - Δ*DS *values. Five of these corresponded to regions likely to be structured (e.g. score < 0.5), so from the energetic point of view described above it is not clear how these changes could affect structure. On the other hand, the observed changes (P→L, D→Y and S→L) could certainly lead to protein malfunction.

The last three examples involved amino acid changes that would increase the structural tendency within a region of disorder. Interestingly, recent work suggests that mutations causing disorder tendencies to change to structure tendencies occur much more often than the reverse [5,19]. Furthermore, a very high fraction of disease-associated mutations mapping to regions of disorder exhibit such tendencies [5] while a significant fraction of these mutations are also associated with the loss of sites of posttranslational modification [20]. Note that posttranslational modifications very commonly occur in disordered regions [1], perhaps much more often than in ordered regions, especially those modifications involving phosphorylation [21,22].

Also note the earlier work cited above indicating that 114/122 mutations in disordered regions proved to be deleterious [12]. Thus, it would be worthwhile to obtain these data and determine the disorder prediction threshold with the best identification of these harmful mutations. Such a new result could then be included in any future work.

## Conclusions

The use of mutation-induced changes in disorder prediction scores, called Δ*DS*, has been studied here. The overall idea is that a significant change in the tendency of a protein region to be structured or disordered could lead to malfunction of such a protein. The initial findings on the data provided by GAW17 give insight with regard to the directions to explore on real data. For example, with real data one could explore whether some particular threshold for Δ*DS *would give significant separation of harmless versus harmful mutations. If the data of reference [18] turns out to be a general finding, or even if only applicable to certain diseases, this observation, the discovery of which came out of this work, certainly points towards important new directions to try.

There are some limitations for the study. Single SNV may not change the disorder properties too much, since changes of disorder score depend more on changes of a segment than an individual mutation. So in our further work, we would try to investigate the combination or pattern of SNVs nearby the high Δ*DS *spot.

## Competing interests

The authors declare that they have no competing interests.

## Authors' contributions

YW and AKD designed the study. AKD, YW, YL, YH and JJ designed and performed the computational modelling and drafted the manuscript. All the authors read and approved the final manuscript.
